# Using status of secondary prevention medications in post-stroke dysphagia patients: time to raise awareness and develop special formulations

**DOI:** 10.1038/s41598-024-66407-0

**Published:** 2024-07-04

**Authors:** Na Yu, Jianhong Yang, Haliza Katas

**Affiliations:** 1 Centre for Drug Delivery Technology and Vaccine (Centric), Faculty of Pharmacy, Universiti Kebangsaan Malaysia, 50300 Kuala Lumpur, Malaysia; 2 Department of Pharmaceutical Preparation, General Hospital of Ningxia Medical University, No. 804, Shengli South Street, Yinchuan, 750004 China; 3 School of Pharmacy, Ningxia Medical University, Yinchuan, Ningxia China

**Keywords:** Stroke, Dysphagia, Secondary prevention, Perception, Patient preference, Stroke, Drug regulation

## Abstract

Post-stroke dysphagia (PSD) is an increasingly common complication of stroke. Despite its intuitively unfavorable impact on secondary prevention medication use, limited awareness is available regarding this issue. Herein, a cross-sectional survey was conducted to determine the current use, patient-perceived needs and preferences for secondary prevention medications among PSD patients. To emphasize the unique context related to dysphagia, we recruited Chinese stroke patients with a duration of less than 5 years. These patients were initially categorized into PSD respondents with and without dysphagia. Among the 3490 eligible respondents, 42.7% reported experiencing dysphagia after stroke. Those PSD respondents were more likely to consume multiple medications and suffer from anticoagulants-associated gastrointestinal bleeding as compared to non-PSD ones (p < 0.001). More crucially, 40.2% of them had frequent difficulty in swallowing pills, 37.1% routinely crushed solid oral dosage forms (SODFs), and 23.5% coughed frequently when taking SODFs. In consequence, 87.4% responded a need for PSD-specific formulations where safe swallowing, easy swallowing, and reduced medication frequency were preferred pharmaceutical factors. These findings demonstrate an unsatisfactory situation and definite needs for PSD patients in using secondary prevention medications. Awareness should be increased to develop PSD-specific formulations for safe and effective secondary prevention.

## Introduction

The increasing number of aging-related chronic disease cases has become a major global public health issue. As one of the most common chronic diseases, stroke remains the third leading cause of death and disability worldwide and the leading cause in China, imposing a severe social and economic burden on individuals, families, and countries^1–3^. Post-stroke dysphagia (PSD), defined as difficulty in swallowing, is a common dysfunctional complication after a stroke, particularly among the elderly^4^. Due to the rapidly growing aging population and increasing morbidity of stroke, PSD incidence during the acute stroke phase has increased to 37–78%^5,6^. Since the improvement in swallowing ability is associated with neurological recovery, up to 50% patients suffer from varying degrees of dysphagia even 6 months after stroke onset or even develop persistent dysphagia^7^. Among the observed outcomes, PSD was strongly correlated with poor prognosis and elevated mortality, mainly because of increased dysphagia-mediated respiratory infections and malnutrition^8–10^. Additionally, however, despite its high prevalence and harmful effects, poor clinical intervention outcomes of PSD are usually due to the unsatisfactory adherence and accessibility to traditional or emerging therapies^4^. Therefore, PSD management is an urgent concern for stroke patients.

Due to the increased respiratory infections rate^11^ and alteration on drug dissolution characteristics in current compensatory measures in clinic^12^, the oral administration of secondary prevention medications is an unsolved and crucial risk factor for PSD management. As highlighted in the international guidelines, long-term prophylaxis for cerebrovascular events with oral anti-thrombotic and lipid-lowering agents for all non-hemorrhagic stroke patients and anti-hypertensive medications for hypertension patients is of vital importance in stroke management^13^. However, dysphagia can impair safe oral intake of not only nutrients, but also solid medications. Moreover, the secondary prevention medications for long-term use are currently available almost exclusively in the form of oral solid dosage forms (SODFs). Previous studies have found that over 40% PSD patients encounter severe difficulties in swallowing SODFs, as they suffer from adverse swallowing reactions, such as bucking, aspiration, or even suffocation^11,14^. Thus, PSD patients require long-term medication, but swallowing pills is difficult and painful. This situation worsens in severe PSD patients, who require multiple medications^15^. To overcome this, crushed SODFs with or without thickened liquids are commonly used for easy swallowing. However, aspiration, poor adherence, medication errors, and potential drug performance changes, particularly for sustained-release SODFs, may occur as unrecognized or overlooked adverse events, posing a threat for the safety, efficacy, and even legality of secondary prevention medications^16,17^.

The patient-centered pharmaceutical design of oral medications is of importance for improving the therapeutic outcomes of patients with special medication needs^18,19^. Developing a disease-specific dosage form based on the needs and preferences of PSD patients is an alternative approach for overcoming medication swallowing difficulties without concerns about safety and lawfulness. However, very limited information and awareness regarding the current use of secondary prevention medications in PSD patients, as well as their needs and preference for appropriate oral dosage forms, is available despite the substantially increased risk of counter-productive or even lethal outcomes triggered by trouble in swallowing pills^11,12,16^. This shows the imperative need for such studies prior to the development of disease-specific oral dosage forms for PSD patients. Moreover, fluid gel, a new formulation concept proposed recently^20,21^, is promising due to its adjustable rheological characteristics for ease of swallowing, as well as its ability to prevent aspiration-mediated pneumonia in elderly dysphagia patients^22^. Accordingly, this study has been designed to clarify the status of secondary prevention medications in PSD patients, as well as their demands and preferences for oral preparations, using a representative population-based survey in China. Furthermore, the acceptability of fluid gel as a disease-specific dosage form for patients has also been determined to offer a reference for future evidence-based pharmaceutical designs.

## Methods

### Sampling strategy

A cross-sectional, web-based, self-reported survey was conducted among the Chinese population between September 1 and October 15, 2022. Since secondary prevention is most urgently needed within 5 years after the first stroke because of the highest risk of recurrence or even death^23^, individuals who have experienced stroke onset not more than 5 years earlier were screened as the initial target population by clearly stating the target audience in the introduction of the survey and asking about the disease course in the first part of the questionnaire. To identify the respondents without dysphagia, the survey was labeled as a general health investigation for post-stroke individuals rather than a dysphagia-focused study. Only respondents who reported dysphagia were invited to complete the entire survey. Those with a history of esophageal or throat cancer were excluded, as these conditions may interfere with dysphagia type and severity assessment. Moreover, since the elderly are the main population with both stroke and dysphagia, geriatric respondents with age-related dysphagia were excluded by inquiring whether significant dysphagia was experienced before stroke onset.

To obtain a representative Chinese population-based sample, participants were recruited by partnering with the survey research firm http://www.wenjuan.com, which is one of the most professional online survey companies in China. They cooperate with participating hospitals nationwide and have population-specific categorized sample library, including stroke patients. The stroke patients in their sample library are those who have undergone imaging diagnosis (e.g., Brain CT, MRI) and have been hospitalized. Additionally, they used the economic regions of China (first-, new first-, second-, and third-tier) as quotas for sampling based on the latest Chinese census data. Survey invitations were distributed to individuals who/whose family members have experienced stroke onset via an online community for stroke patients. The target participant, who clicked on the survey link, was diverted to our homepage and digital informed consent was collected before participating. They would be brought to our questionnaire page only if they understood our study purpose and agreed to participate by clicking “I agree” at the end of the consent form. During the data cleaning process, the response authenticity was verified by checking their cookies, IP, email addresses, and disease diagnosis certificates. Any abnormal responses, including answering questions casually, inconsistently, or repeatedly, were automatically eliminated. Ethics approval was obtained from the local ethics committees of the Universiti Kebangsaan Malaysia and Ningxia Medical University (JEP-2022-577 and 2022-61) as the study was part of a cooperative project between them. All methods in this study have been performed in accordance with the Declaration of Helsinki.

### Survey instruments

Due to the extensive literature review and input from experts in the field of stroke care, we developed a series of survey items to improve our understanding of the current situation as well as the needs and preferences for secondary prevention medications in PSD patients. The questionnaire comprised the following four sections (Supplementary File 1).

In the first section, participant demographic information, including sex, age, home town, living status, employment status, educational level, and clinical stroke characteristics (course and subtype), were collected. Secondary prevention medication use was surveyed by providing a list of almost all currently available medications, which were categorized as anti-thrombotic, anti-lipemic, anti-hypertensive, and anti-hyperglycemic medications. To further investigate medication compliance, the respondents were then asked to state the using situation of each category based on following options, where various degrees of medication compliance were defined as regularly, discontinued ≤ 7 days, discontinued 8–30 days, discontinued 31–60 days, and discontinued > 60 days. For the first purpose of this study, eight gastrointestinal (GI) symptoms, including dysphagia^24^, were listed as a blinded screener to categorize all initial participants into two groups: those who experienced dysphagia after stroke and those who did not experience dysphagia since stroke onset. Only the group with dysphagia completed the remaining survey.

In the second section, dysphagia severity and compensatory behaviors were assessed by employing the dysphagia domain of the National Institutes of Health (NIH) Patient-Reported Outcomes Measurement Information System (PROMIS^®^) GI symptom scales^24,25^. In particular, the information about pill crushing or cutting frequency (as compensatory behaviors for ease of swallowing) as well as suffering a cough when taking SODFs on a scale from “never” to “always” were also collected. Furthermore, questions were also set to elucidate their personal perception of dysphagia severity and its influence on medication intake, for verifying the NIH PROMIS^®^-based results.

In the third section, the extent of ease for directly swallowing available secondary prevention medications (without breaking or crushing) as well as the necessity of developing new oral drug dosage forms were rated on a 5-point scale from “strongly disagree” to “strongly agree.” To provide evidence for future patient-centered pharmaceutical design, the preference of respondents with PSD for secondary prevention medications, including dosage form, release behavior, and need-based factors for PSD-specific dosage forms, was clarified subsequently.

The fluid gel, as verified in our previous study, promises to be an appropriate dosage form for senile dysphagia patients. Therefore, we determined the perception and acceptability of fluid gels as a novel dosage form for PSD patients in the last section of our survey instrument. A straightforward description of the fluid gel was provided before the questions to draw a clear picture for the respondents. Finally, the perceived advantages and risks of fluid gels were investigated by listing all possible aspects to choose and rank.

### Statistical analyses

The calculated minimum sample size for this study is 2038, relevant calculation details are provided in Supplementary File 1. Considering funding and potency, the survey sample size was set at approximately 3500. All analyses were performed using Stata MP 17.0 software. Demographic characteristics and survey responses are summarized as frequencies and percentages using descriptive statistics. To compare the use of secondary prevention medications in the groups with and without dysphagia, the chi-square test and Student’s *t*-test were used to analyze categorical and continuous variables, respectively. Statistical significance was set at a two-tailed p < 0.05. For correlation analysis, logistic regression models adjusted for demographic variables were used, with the results presented as odds ratios (ORs) and p-values under the corresponding 95% confidence intervals (CIs). Dysphagia severity was assessed by calculating the PROMIS^®^ score as a percentile and graded by quartile as reported previously^24^. For multiple-select survey items, further ranking was determined and analyzed by calculating the average rank score based on a weight assignment method and Pareto charts were employed to identify the dominant matter.

### Ethical approval

The study was approved by the Research Ethics Committee of both Universiti Kebangsaan Malaysia (JEP-2022-577) and Ningxia Medical University (2022-61).

### Patient consent

Informed consent was obtained from all participants included in the study in a electronic form prior to their inclusion in the study.

## Results

### Study sample and demographics

Overall, 43,958 recipients were invited to complete the survey. Only 13,741 participants responded to the survey (response rate: 31.3%). Among them, we excluded 10,233 respondents with an abnormal response (those who answered questions incompletely, casually, inconsistently, repeatedly, or quickly, and those who failed to pass the authenticity verification), 10 respondents with previous esophageal or throat cancer diagnosis, and 8 with dysphagia prior to stroke onset. Ultimately, 3490 responses were included in the analysis.

The demographic and clinical characteristics of the final cohort are summarized in Table 1. Age was clustered around 51–70 years (65.5%), with a male predominance (65.0%). Among the 3490 eligible respondents, 1490 (42.7%) reported experiencing dysphagia after stroke. Compared to the respondents without difficulty in swallowing (n = 2000), those with dysphagia were male, older, had a lower educational level, and had a longer course (p < 0.05). Moreover, the respondents with dysphagia were less likely to participate in the labor force (9.7% vs. 20.8%, p < 0.001) and more likely to have physical disabilities (2.8% vs. 0.0%, p < 0.001). Moreover, the constituent ratios of clinical stroke subtypes (hemorrhagic, ischemic, and undetermined) were also significantly different between the two groups (p < 0.001), reflecting due to the high proportion of hemorrhagic stroke patients in the dysphagia group (30.9% vs. 16.6%, p < 0.001).

**Table 1:** Baseline demographic and clinical characteristics of cohort.

Characteristics^a^	All participants (n = 3490)	With post-stroke dysphagia		
		Yes (n = 1490)	No (n = 2000)	p^b^
Sex				
Male	2267 (65.0)	1019 (68.4)	1248 (62.4)	**< 0.001**
Female	1223 (35.0)	471 (31.6)	752 (37.6)	
Age, years				
21–30	2 (0.1)	0 (0.0)	2 (0.1)	**< 0.001**
31–40	55 (1.6)	3 (0.2)	52 (2.6)	
41–50	696 (19.9)	250 (16.8)	446 (22.3)	
51–60	1096 (31.4)	454 (30.5)	642 (32.0)	
61–70	1191 (34.1)	531 (35.6)	660 (33.0)	
71–80	406 (11.6)	211 (14.2)	195 (9.8)	
81–90	44 (1.3)	41 (2.7)	3 (0.2)	
City				
First-tier cities	832 (23.8)	354 (23.8)	478 (23.9)	0.999
New first-tier cities	1023 (29.3)	438 (29.4)	585 (29.2)	
Second-tier cities	960 (27.6)	409 (27.4)	551 (27.6)	
Third-tier cities	675 (19.3)	289 (19.4)	386 (19.3)	
Living status				
Live with family	3393 (97.2)	1458 (97.9)	1935 (96.8)	**0.030**
Live alone	64 (1.8)	17 (1.1)	47 (2.3)	
Live in a nursing institution	33 (1.0)	15 (1.0)	18 (0.9)	
Working status				
Working (full-time or part time)	561 (16.1)	145 (9.7)	416 (20.8)	**< 0.001**
On leave from work	220 (6.3)	99 (6.6)	121 (6.0)	
Not working	2669 (76.5)	1206 (80.9)	1463 (73.2)	
On disability	40 (1.1)	40 (2.8)	0 (0.0)	
Education level				
Elementary education or below	533 (15.3)	266 (17.9)	267 (13.3)	**0.001**
Secondary education	2040 (58.4)	833 (55.9)	1207 (60.4)	
Higher education	917 (26.3)	391 (26.2)	526 (26.3)	
Stroke course				
≤ 1 years	898 (25.7)	223 (15.0)	675 (33.8)	**< 0.001**
1–2 years	1499 (43.0)	605 (40.6)	894 (44.7)	
2–5 years	1093 (31.3)	662 (44.4)	431 (21.5)	
Stroke subtype				
Hemorrhagic stroke	792 (22.7)	461 (30.9)	331 (16.6)	**< 0.001**
Ischemic stroke	2589 (74.2)	1029 (69.1)	1560 (78.0)	
Undetermined	109 (3.1)	0 (0.0)	109 (5.4)	

^a^Data are presented as n (%); percentages represent the distribution of categorical variables among all participants or the split between participants with and without dysphagia.

^b^p are from the chi-square test for group comparisons. Statistically significant p is indicated in bold.

### Secondary prevention medication use: medication consumed and compliance

Most of the 3490 eligible respondents consumed at least one secondary stroke prevention medication (89.0%, 59.8%, 49.9%, 24.0% on anti-thrombotic, anti-hypertensive, anti-lipemic, and anti-hyperglycemic medications, respectively; Table 2). A significantly high percentage of respondents with PSD consumed secondary prevention medications (anti-thrombotic: 97.9% PSD vs. 82.4% non-PSD, anti-hypertensive: 75% PSD vs. 48.4% non-PSD, anti-lipemic: 72.3% PSD vs. 33.4% non-PSD, anti-hyperglycemic: 32.3% PSD vs. 17.9% non-PSD; p < 0.001). Among these medications, aspirin, atorvastatin, clopidogrel, and nifedipine were most frequently used in the entire study panel and both groups. Moreover, participants with PSD were likely to consume more than two medications concurrently (76.4% PSD vs. 32% non-PSD, p < 0.001), experience anti-thrombotic medication-mediated GI ulcers or bleeding (24.2% PSD vs. 15.9% non-PSD, p < 0.001), and experience helpless discontinuation of anti-thrombotic medications due to GI ulcers or bleeding (17.1% PSD vs. 13.6% non-PSD, p = 0.004).

**Table 2:** The use of secondary prevention medications after stroke.

Items^a^	All participants (n = 3490)	With post-stroke dysphagia		P value^b^
		Yes (n = 1490)	No (n = 2000)	
Antithrombotic				
Total	3107 (89.0)	1458 (97.9)	1649 (82.4)	**< 0.001**
Aspirin	2552 (73.1)	1226 (82.3)	1326 (66.3)	**< 0.001**
Clopidogrel	1224 (35.1)	660 (44.3)	564 (28.2)	**< 0.001**
Others	92 (2.6)	14 (0.9)	78 (3.9)	**< 0.001**
Antilipemic				
Total	1741 (49.9)	1078 (72.3)	663 (33.2)	**< 0.001**
Atorvastatin	1200 (34.4)	724 (48.6)	476 (23.8)	**< 0.001**
Rosuvastatin	397 (11.4)	242 (16.2)	155 (7.8)	**< 0.001**
Others	144 (4.1)	112 (7.5)	32 (1.6)	**< 0.001**
Antihypertensive				
Total	2086 (59.8)	1117 (75.0)	969 (48.4)	**< 0.001**
Nifedipine	941 (27.0)	492 (33.0)	449 (22.4)	**< 0.001**
Amlodipine	617 (17.7)	357 (24.0)	260 (13.0)	**< 0.001**
Others	644 (18.5)	320 (21.5)	324 (16.2)	**< 0.001**
Antihyperglycemic				
Total	839 (24.0)	481 (32.3)	358 (17.9)	**< 0.001**
Metformin	351 (10.1)	184 (12.3)	167 (8.4)	**< 0.001**
Acarbose	248 (7.1)	161 (10.8)	87 (4.4)	**< 0.001**
Others	268 (7.7)	106 (5.3)	162 (10.9)	** < 0.001**
Number of medications used				
Taking ≤ 2 medications concurrently	1711 (49.0)	352 (23.6)	1359 (68.0)	**< 0.001**
Taking > 2 medications concurrently	1779 (51.0)	1138 (76.4)	641 (32.0)	
Experienced gastrointestinal ulcers or bleeding caused by antithrombotic medications				
Yes	679 (19.5)	361 (24.2)	318 (15.9)	**< 0.001**
No	2811 (80.5)	1129 (75.8)	1682 (84.1)	
Inability to take antithrombotic medications normally due to gastrointestinal ulcers or bleeding				
Yes	526 (15.1)	255 (17.1)	271 (13.6)	**0.004**
No	2964 (84.9)	1235 (82.9)	1729 (86.4)	

^a^Data are presented as n (%); percentages represent the proportion of participants conforming to each item among all study samples or the split between groups. Items listed as “total” represent the proportion of participants who consumed corresponding class of drugs.

^b^p are from the chi-square test; for group comparisons, statistically significant p-values are in bold.

**Figure 1:**
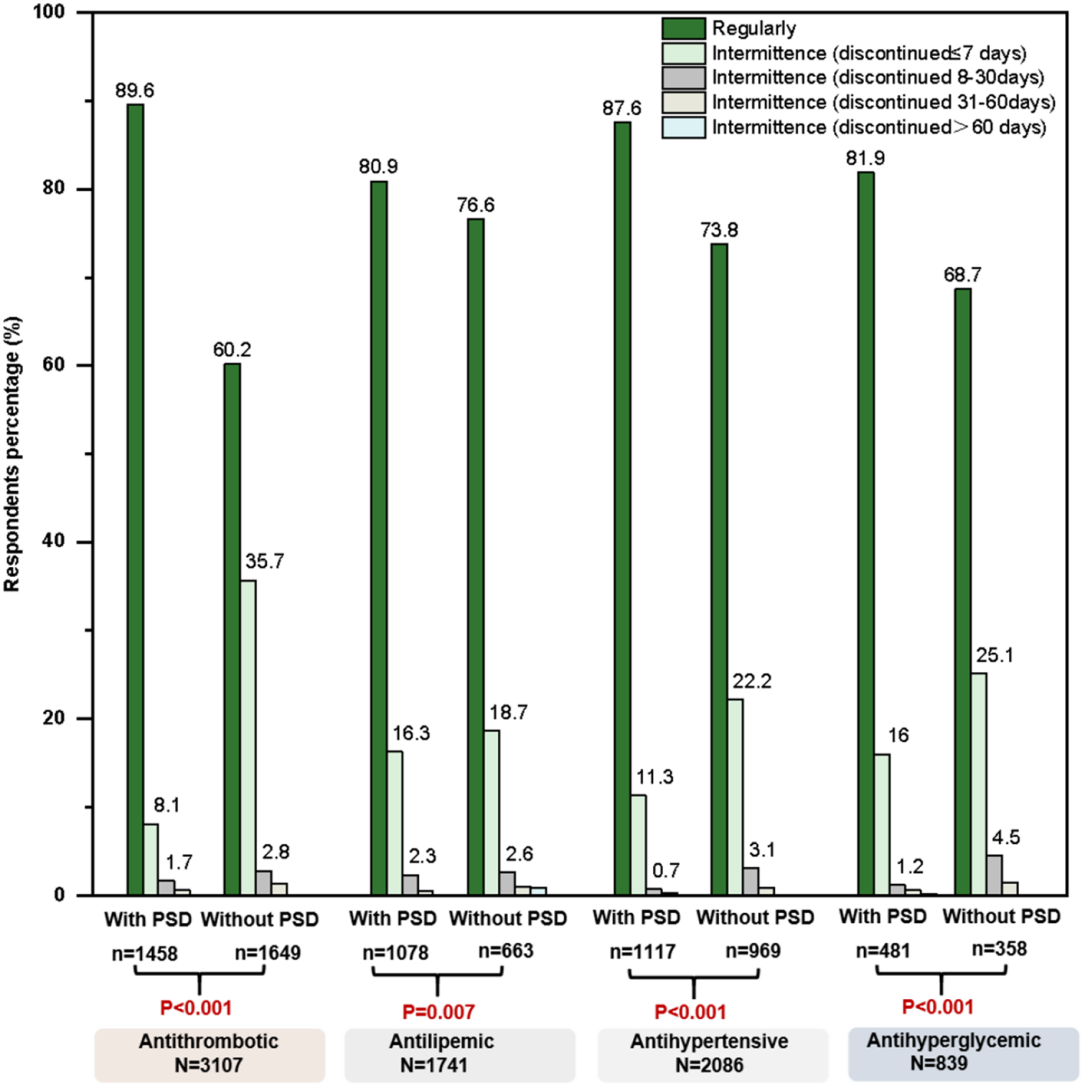
The compliance for secondary prevention medications. The profile of drug compliance was classified as regularly and varying degrees of discontinuity (discontinued ≤ 7 days, 8–30 days, 31–60 days, and > 60 days). p-values between respondents with and without post-stroke dysphagia (PSD) were determined by chi-square test.

**Figure 2:**
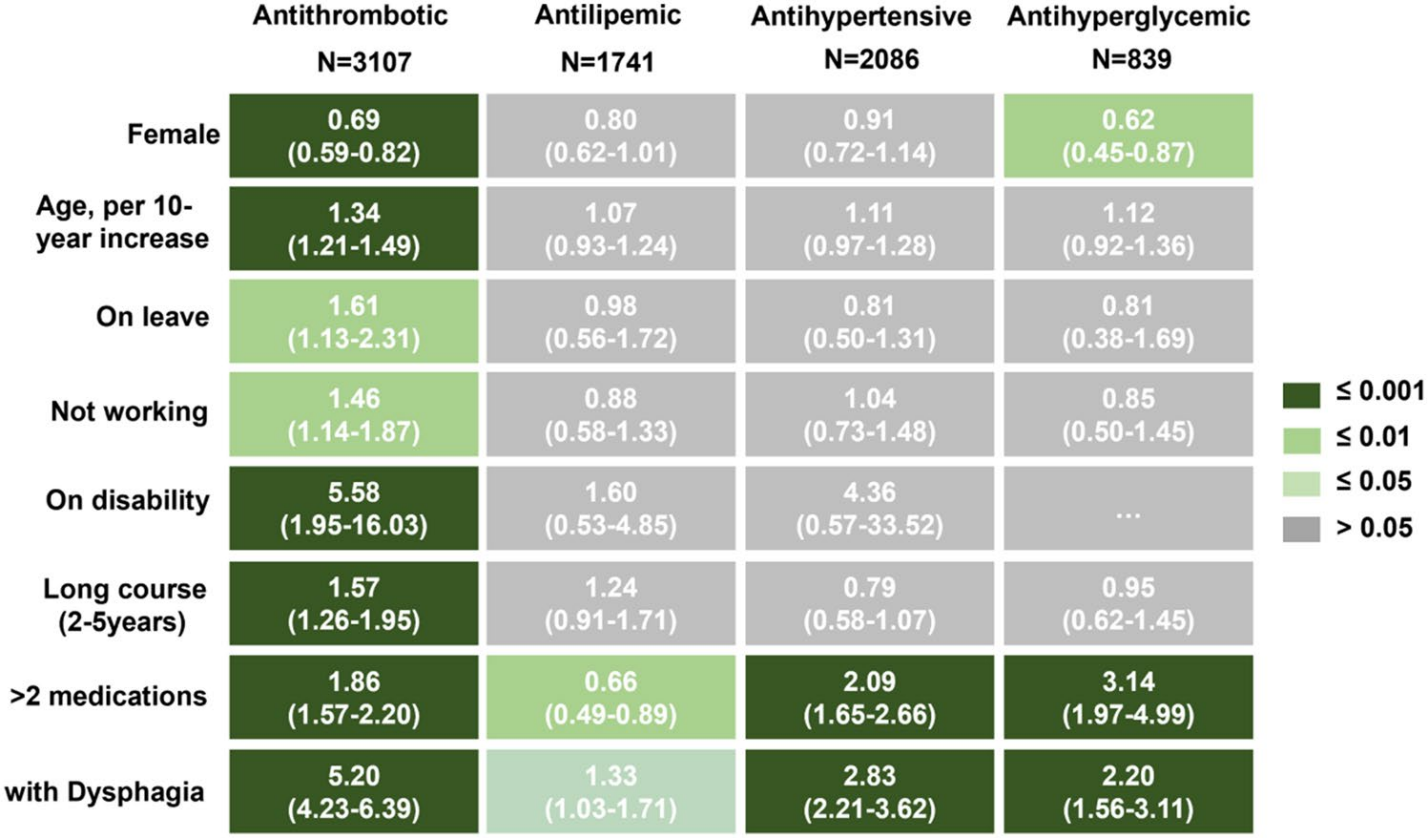
Factors associated with compliance of secondary prevention medications. Adjusted multiple logistic regression for the associated of various factors including dysphagia with regular consumption of different class of medications was performed. Each factor adjusted for sex, age, working status, education level, stroke course, and stroke subtype.

Over 70% stroke respondents reported that they adhered to (responded as “regularly”) the secondary prevention medications (74.0%, 76.3%, 79.3%, and 81.2% for anti-thrombotic, anti-hyperglycemic, anti-lipemic, and anti-hypertensive medications, respectively; Supplementary File 2 Table 1). However, the effects of the anti-thrombotic, anti-hypertensive, and anti-hyperglycemic medications were significantly different (p < 0.001), while the anti-lipemic effects were relatively less significantly different between the two groups (p = 0.007; Fig. 1). Specifically, respondents with PSD were more likely to consume secondary prevention medications regularly than those without PSD (89.6% vs. 60.2%, 87.6% vs. 73.8%, 81.9% vs. 68.7%, and 80.9% vs. 76.6% for anti-thrombotic, anti-hypertensive, anti-hyperglycemic, and anti-lipemic medications, respectively). Further multivariate analysis showed similar associations for anti-thrombotic (adjusted OR [95% CI]: 5.20 [4.23–6.39], p ≤ 0.001), anti-hypertensive (2.83 [2.21–3.62], p ≤ 0.001), anti-hyperglycemic (2.20 [1.56–3.11], p ≤ 0.001), and anti-lipemic (1.33 [1.03–1.71], p ≤ 0.05) medications (Fig. 2; Supplementary File 2 Table 2). Compared with participants who consumed only one or two medications, those consumed more than two medications were more likely to have a good compliance for anti-thrombotic (1.86 [1.57–2.20], p ≤ 0.001), anti-hypertensive (2.09 [1.65–2.66], p ≤ 0.001), and anti-hyperglycemic (3.14 [1.56–3.11], p ≤ 0.001) medications, despite a weak opposite association for anti-lipemic medications (0.66 [0.49–0.89], p ≤ 0.01). Moreover, participants with good compliance for anti-thrombotic medications were more likely males (0.69 [0.59–0.82], p ≤ 0.001), elderly (1.34 [1.21–1.49], p ≤ 0.001), disabled (5.58 [1.95–16.03], p ≤ 0.001), on leave (1.61 [1.13–2.31], p ≤ 0.01), or not working (1.46 [1.14–1.87], p ≤ 0.01).

### Dysphagia severity measured by NIH PROMIS^®^ and its influence on medication intake

**Figure 3:**
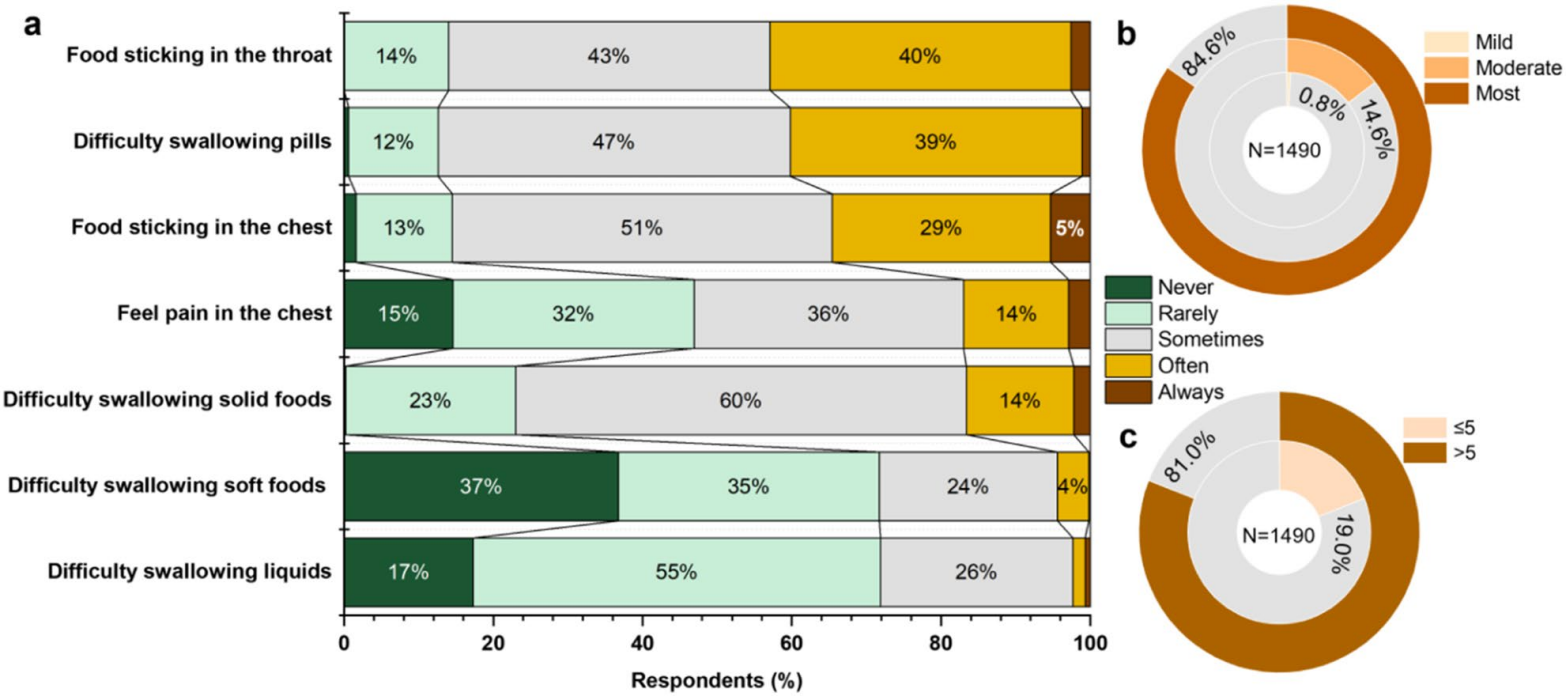
Dysphagia severity as measured by National Institutes of Health (NIH) Patient-Reported Outcomes Measurement Information System (PROMIS^®^) scales. (**a**) Responses of respondents with post-stroke dysphagia (PSD) for each PROMIS^®^ item. (**b**) Distribution of the respondents with PSD with varying severity of dysphagia symptoms classified by quartile according to the results of a previous study (the first, second, third, and forth quartile represent least, mildly, moderately, and most symptomatic dysphagia, respectively)^24^. (**c**) Self-perception of the respondents with PSD on severity of pain with swallowing surveyed according to a 0–10 pain scale.

As measured using the NIH PROMIS^®^, respondents with PSD experienced different levels of dysphagia symptoms within the past week (Fig. 3a). Among these, “Food sticking in the throat,” “difficulty swallowing pills,” and “food sticking in the chest” were the most common symptoms (responded as “often” and “always”) in over one third respondents with PSD.

Approximately 40% participants experienced frequent difficulties in swallowing pills. Based on the responses to these seven items, dysphagia severity was further graded by calculating the centile score and stratifying them according to quartiles. The median percentile score on the scale among the respondents with PSD was 88 (interquartile range [IQR], 12). Of the total 1490 participants with PSD, 1260 (84.6%), 218 (14.6%), and only 12 (0.8%) suffered from “most symptomatic,” “moderate symptomatic,” and “mild symptomatic” dysphagia, respectively (at fourth, third, and second quartiles, respectively; Fig. 3b). Furthermore, using a pain scale of 0 (no pain with swallowing) to 10 (severe pain with swallowing), 1260 (81%) rated their pain with swallowing as > 5, which were similar to the proportion of “most symptomatic” respondents (Fig. 3b).

**Figure 4:**
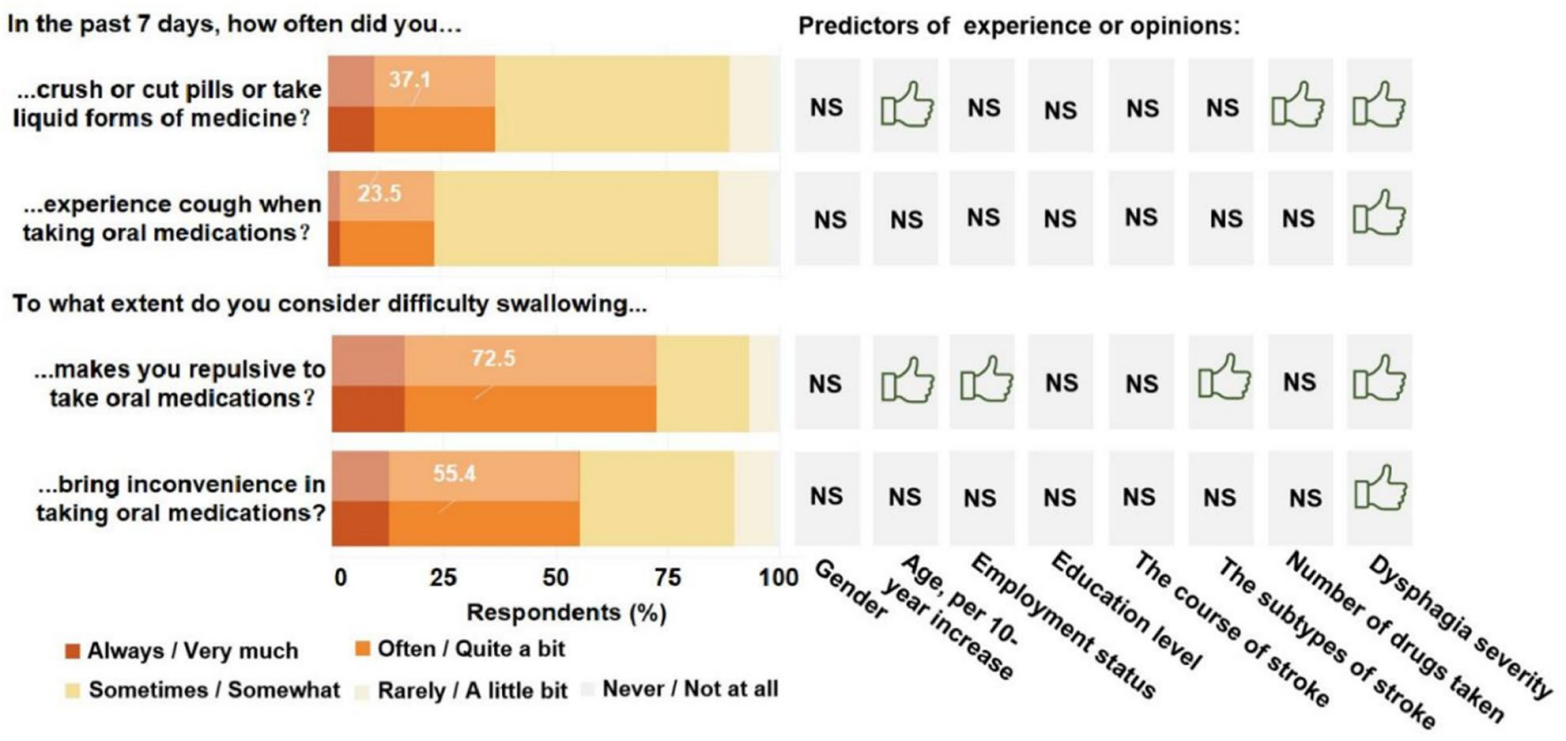
Major compensatory behaviors, adverse effect, and patient-perceived influence of dysphagia on oral medication intake. Associated factors for each item were analyzed by adjusted multiple logistic regression. Each factor adjusted for sex, age, working status, education level, stroke course, stroke subtypes, number of medications used, and dysphagia National Institutes of Health (NIH) Patient-Reported Outcomes Measurement Information System (PROMIS^®^) score as the quartile. Green thumbs represent significant positive correlation (p ≤ 0.05) and “NS” represents non-significant correlation.

To cope with the above varying degrees of symptoms, respondents with PSD were engaged in diverse compensatory behaviors for dysphagia at different frequencies (Supplementary File 2 Table 3). Particularly, 553 (37.1%) respondents with PSD crushed or cut SODFs or consumed liquid medicine forms frequently (responded as “often” and “always”) as compensatory behaviors for the ease of swallowing pills, and subsequently 351 (23.5%) experienced coughs frequently when taking SODFs (Fig. 4). In addition, 825 (55.4%) and 1081 (72.5%) respondents with PSD perceived that dysphagia bring inconvenience and makes them repulsive to oral medications (responded as “quite a bit” and “very much,” respectively). Following further multivariable regression, respondents with “most symptomatic” dysphagia were likely to use crushed SODFs, experience cough, and believe that difficulty in swallowing develops repellent emotion and inconvenience in taking SODFs (Fig. 4; Supplementary File 2 Table 4, Table 5, Table 6, Table 7).

### Needs and preference toward preparations of secondary prevention medications

**Figure 5:**
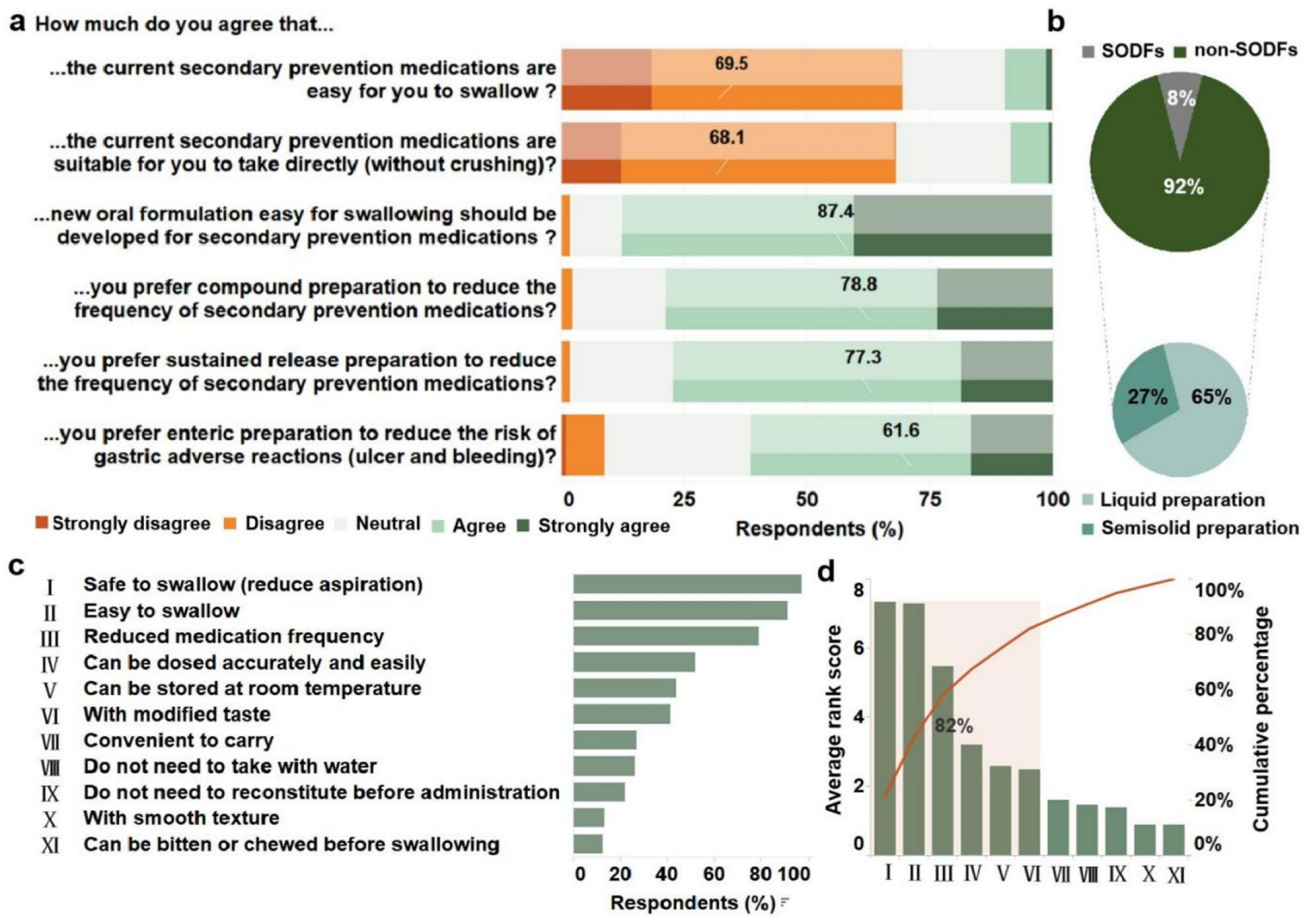
Needs and preference of respondents with post-stroke dysphagia (PSD) toward secondary prevention medications. (**a**) Responses of respondents with PSD for major survey items regarding needs and preference for secondary prevention medications. (**b**) Distribution of the responses for preference of oral dosage forms. (**c**) Self-perception of essential factors for PSD-specific oral dosage form. (**d**) Primary factors determined by Pareto charts based on average rank score. To calculate the average rank score of each above-mentioned essential factor, ranking of chosen factors was asked, and weight assignment method was employed.

Most respondents with PSD (n = 1036 [69.5%]) felt that the current secondary prevention medications are not easy to swallow (responded as “strongly disagree” and “disagree,” Fig. 5a). This was further confirmed by a similar proportion of participants (n = 1031 [68.1%]) who responded with a definite perception of the inapplicability of these drugs to be consumed directly without breaking or crushing. When asked about the need of developing new easily swallowable oral dosage forms, much higher proportion of respondents with PSD (n = 1302 [87.4%]) replied affirmatively (responded as “strongly agree” and “agree,” Fig. 5a). Furthermore, 1370 (92.0%) respondents with PSD preferred non-SODFs, including oral liquid formulations (967 [64.9%]) and semi-solid formulations (403 [27.1%]), whereas only 120 (8.0%) preferred SODFs (Fig. 5b). Additionally, sustained-release preparations (n = 1151 [77.3%]) and enteric-release preparations (n = 918 [61.6%]) were also preferred for the combination of two or more medications (n = 1173 [78.7%]). Specifically, “safe to swallow (avoid aspiration)” was the most common response in the subsequent investigation on self-perception of essential factors for a PSD-specific dosage form (97.4%), followed by “easy to swallow” (91.5%), and “reduced medication frequency” (79.2%; Fig. 5c). Moreover, “safe to swallow,” “easy to swallow,” “reduced medication frequency,” “can be dosed accurately and easily,” “with modified taste,” and “can be stored at room temperature” were preferred primary factors for PSD-specific dosage form according to the Pareto Principle (Fig. 5d).

### Acceptability of fluid gels as PSD-specific dosage form

**Figure 6:**
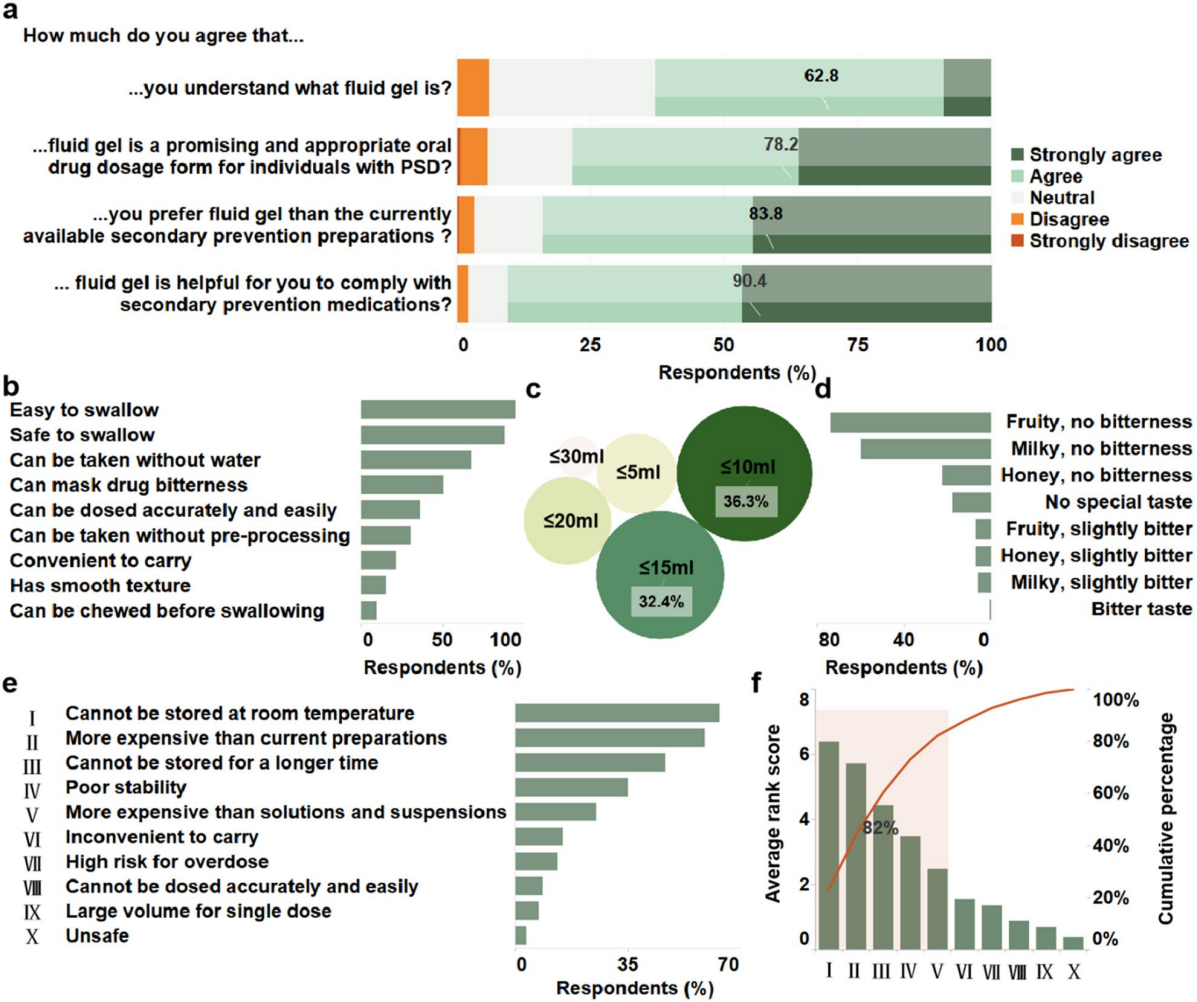
Acceptability of fluid gels as a post-stroke dysphagia (PSD)-specific dosage form. (**a**) Responses of respondents with PSD for acceptability-relevant survey items. (**b**) Perceived advantages of fluid gel as PSD-specific oral dosage form. (**c**) Acceptable volume of fluid gel for a single dose. (**d**) Acceptable taste of fluid gel. (**e**) Perceived risks of fluid gel as PSD-specific oral dosage form. (**f**) Primary risks determined by Pareto charts based on average rank score.

Almost two-third respondents with PSD (936 [62.8%]) clearly understood the concept of fluid gel (responded as “strongly agree” and “agree”) after receiving a straightforward description (Fig. 6a). Accordingly, most respondents with PSD believed that fluid gel is a promising PSD-specific oral drug dosage form (1165 [78.2%]) that favors compliance with secondary prevention medications (1346 [90.4%]), and preferred it over the currently available preparations if developed as a usable pharmaceutical formulation (1249 [83.8%]).

Furthermore, swallowing ease and safety, no need to drink water when and after taking medicines, and the ability to mask bitterness were most commonly perceived as the advantages of fluid gel with over 50% response frequency (Fig. 6b). In addition, a single 10–15 mL dose (1024 [68.7%]) and a fruity or milky taste without bitterness (1100 [73.8%] or 888 [59.6%], respectively) were the most universally accepted advantages for developing fluid gels as PSD-specific dosage forms (Fig. 6c,d). Finally, in the investigation on perceived risks of fluid gel, “cannot be stored at room temperature,” “more expensive than current solid dosage forms,” “cannot be stored for a longer time,” “formulation instability,” and “more expensive than solutions and suspensions” were the important aspects that worried more than 25% respondents with PSD (Fig. 6e). Subsequent Pareto analysis further confirmed these five factors as the primary risks that must be addressed and surmounted in the future development of PSD-specific fluid gels (Fig. 6f).

## Discussion

In this study, we systematically described the current situation as well as self-perceived demands and preferences for secondary prevention medications in PSD patients for the first time. The initial results show that PSD patients tend to be older, less educated, have a longer disease course, underworked, and more disabled than those without dysphagia. Additionally, they are more likely to consume multiple medications, suffer from anti-thrombotic medication-associated upper GI ulcer or bleeding, as well as be caught in dilemma of having to stop anti-thrombotic medications, particularly aspirin, because of severe GI bleeding. Moreover, a significant proportion of PSD patients suffered from frequent difficulty in swallowing pills (40.2%), crushed SODFs as a compensatory maneuver for difficulty in swallowing pills (37.1%), and were plagued by frequent coughing when taking SODFs (23.5%), which correlated positively with the NIH PROMIS^®^ scale-based severity of dysphagia. These findings provide substantial evidence that PSD patients confront more challenges and hidden risks when using current secondary prevention medications than post-stroke patients without trouble in swallowing, highlighting the urgent need for PSD patients as a special community to improve the actuality of using secondary prevention medications. Equally significant, further results indicate a definite need (confirmed by 87.4% respondents with PSD) and specific preferences for preparing secondary prevention medications, and meanwhile, encourage the acceptability (confirmed by 83.8% respondents with PSD) of fluid gel as a PSD-specific dosage form, providing patient-centered evidence for improving the above-highlighted situation from a pharmaceutical perspective.

Previous studies on PSD patients mainly focused on the incidence^4–6^, diagnosis^4,5,26^, consequence^7–10^, risk factors^27,28^, treatment^29–33^, aspiration^34,35^, and prognosis^7,32,36,37^. Very little information concerning the potential challenges in secondary prevention medication administration is available. However, despite considerable strides by neuroscientists in the last few years to identify, characterize, prevent, and improve or even regain the ability to swallow, the clinical outcomes have been limited because of various barriers, including poor accessibility, adherence, and effectiveness of the reported intervention means^4^. As we have mentioned, 37–78% acute stroke patients experience dysphagia during hospital stay^5,6^, and 50% initially diagnosed PSD patients still have persistent dysphagia at hospital discharge^37^ or even 6 months after stroke onset^7^. Likewise, over 40% of the 3490 post-stroke respondents in our cross-sectional investigation were identified as PSD patients in an inclusion criterion of having suffering some degree of dysphagia after stroke and up to 80% of them still suffered from “most symptomatic” dysphagia as measured by NIH PROMIS^®^ scales. Most importantly, we also demonstrate that a considerable proportion of patients face problem in using secondary prevention medications, which serves as a forceful complement and reinforces the results of previous limited studies^11,14^. Admittedly, in addition to reducing morbidity and improving the outcomes of dysphagia, current disadvantageous situations should be overcome to safely and effectively use secondary prevention medications before regaining the capacity of swallowing is of equal significance for PSD patients.

As stated earlier, developing PSD-specific oral dosage forms in a patient-centered pharmaceutical design based upon needs and preferences^18,19^ of PSD patients is a promising approach to improve the current situations discussed above. In this regard, our study also sheds light on the patient-centered needs and preferences toward oral dosage forms for secondary prevention medications. As expected, dysphagia-specific preparations as non-SODFs, which are mainly characterized by low aspiration risk, ease of swallowing, and reduced medication frequency, are universally in demand among PSD patients (affirmed by no less than 80% respondents with PSD). The total positive response on suffering difficulty swallowing pills, applying crushed SODFs, and experiencing cough when taking SODFs (respond as “sometimes,” “often” and “always”) also echoes this, indicating that even if some PSD patients encounter dysphagia-related problems less frequently, they perceived a need for PSD-specific preparations. Additionally, the preference of the pharmaceutical design, sustained-release (to reduce medication frequency) and enteric-release (to avoid anti-platelet-related GI adverse effects), of combined delivery were similar. Overall, these findings provide an important basis and reference for the patient-centered and evidence-based pharmaceutical design of PSD-specific preparations.

Fluid gel, a novel material with a jelly-like appearance and distinct rheological nature, which favors swallowing^20–22^ and is mainly used in the food industry^38^, was a potential alternative form for PSD-specific preparations in terms of self-perceived acceptability. It is noteworthy that in this study, PSD patients responded with a positive perception and high acceptability of fluid gels as dysphagia-specific oral dosage forms than the general public in our previous related study^39^. This may help to corroborate the fact that easily swallowed preparations are urgently needed for PSD patients. Furthermore, the essential factors, acceptable taste, and single dose volume, as well as potential advantages and risks, were elucidated from a patient-perceived perspective, providing comprehensive evidence for further fluid gel development as PSD-specific preparations. Therefore, tailoring fluid gels according to this evidence will probably become an important booster that facilitates the translation of our above-discussed assumption on improving the current situations faced by PSD patients when using secondary prevention medication in practice.

Finally, our survey revealed an excellent compliance with secondary prevention medications in PSD patients. Despite a consistent self-reported, post-stroke medication adherence rate with that reported in previous study (74.0–82.1% in our study vs. 73.0–77.7% in other reports)^40,41^, it was significantly higher in the PSD group than that in the non-PSD group, which is contrary to our common sense-based expectation. Combined with multivariate analysis, we showed that PSD is strongly associated with medication compliance instead, due to the difficulty in swallowing pills. Considering that a significantly higher proportion of respondents were disabled (2.8% vs. 0.0%) and consumed more than two medications (76.4% vs. 32.0%), as well as a lower proportion of normal workers (9.7% vs. 20.8%) in the PSD group, we speculate that this counterintuitive result might be mainly due to the stronger belief in taking medicine in the PSD population, which is inspired by an intensive willingness to alleviate pain due to severe post-stroke complications, including dysphagia. Strong medication beliefs, as suggested by recent studies^42–44^, are strongly associated with medication adherence in stroke patients, partly supporting our inference. Additionally, we corroborated the findings of other related studies on the positive correlation of old age and male sex with medication compliance in stroke patients^43–45^, further enhancing the credibility of our study on the counterintuitive results discussed above.

### Strengths and limitations

This is the first population-based study specifically targeting PSD patients and focuses on their challenges, as well as patient-perceived demands in using oral secondary prevention medications. Moreover, we employed stroke patients without dysphagia as a contrast to elucidate the situation faced by PSD patients more compellingly.

However, despite these strengths, which favor in obtaining instructive findings, our study has several limitations. First, our efforts to locate an available nationwide stroke register system in China for acquiring a population-based sample were unsuccessful, which would have allowed us to conduct rigorous research. To remedy this defect, we partnered with a professional survey research firm to identify and include target participants through a country-wide online stroke patient community. We also labeled our questionnaire as a general health investigation, and then employed eight GI symptoms as blinded screeners to identify PSD patients. The screened PSD respondents were further verified using the NIH PROMIS^®^ scales, which are validated methods to assess dysphagia^24,25^. Second, we did not use quotas for age and sex during sampling due to implementation difficulties, which could be a potential concern about the generalizability of our results. Nonetheless, we used age and tenderness as adjusted factors in the multivariate analysis to minimize the impact of using statistical methods. In addition, we only investigated the needs and preferences of PSD respondents, while participants without dysphagia were asked to conclude the survey earlier, after completing the first section. This could be another limitation of our study, because we neglected the fact that the findings regarding need and preference would be highly reliable if relevant comparable results were also obtained. Last but not least, stroke patients with other comorbid neurological diseases were not excluded, which may lead to some false positive results. However, this impact will not be decisive, as these patients are likely to face more severe swallowing issues that need to be raised awareness of and addressed.

Finally, the limitations of our study include inherent drawbacks to survey studies. Specifically, the generalizability of the findings may be limited by our cross-sectional, self-administered, web-based mode of implementation, low response rate, and subjective and experiential differences between individuals. In the future, a longitudinal follow-up study combined with cross-sectional face-to-face interviews may provide insights into the challenges and corresponding solutions in using oral secondary prevention medications among PSD patients. Overall, despite these limitations, our available data indicate the hidden hazards and challenges in the current situation and underline the urgency and necessity of developing PSD-specific preparations.

## Conclusions

In conclusion, our population-based survey on 3490 Chinese stroke patients suggests that PSD patients face grave situations with dysphagia-mediated challenges and potential risks when using current secondary prevention medications. Consequently, the urgent need and definite preferences for specific preparations that facilitate safe swallowing are universally perceived among PSD patients. Therefore, the patient-centered development of PSD-specific preparations is a promising strategy to improve the current situation. From a pharmaceutical perspective, the fluid gel is an alternative dosage form for PSD-specific preparations, with encouraging patient-perceived acceptance. In summary, it is time to raise awareness and put forward solutions to improve the current situation faced by PSD patients in using oral secondary prevention medications. Future research is urgently needed to shed light on the challenges and corresponding solutions as well as develop PSD-specific preparations for safe and effective secondary prevention.

### Supplementary Information

Supplementary Information.

## Data Availability

The data that support the findings of this manuscript are available from the corresponding author on reasonable request.
