# Supplementary material for: Using status of secondary prevention medications in post-stroke dysphagia patients: time to raise awareness and develop special formulations
Source: Sci Rep. 2024 Jul 4;14:15377. doi: 10.1038/s41598-024-66407-0 (PMC11224274; doi:10.1038/s41598-024-66407-0)
Supplement: Supplementary file 1 — Supplementary Information. [file 41598_2024_66407_MOESM1_ESM.pdf]

Using status of secondary prevention medications in post-stroke dysphagia patients: Time to raise awareness and develop special formulations

Na Yu[1,2], Jianhong Yang[3], Haliza Katas[1,*]

Supplementary File 1

General Health Questionnaire for Stroke patients

Introduction

Target audience

Individuals who/whose family members have experienced the onset of stroke not more than 5 years. (You can fill it out on behalf of your family)

What is the purpose of the Survey?

This is a survey investigating the health status of individuals after stroke, aiming to provide reference for improving their prognosis and life quality.

Where do the questions come from?

The questions in this study were formulated by a dedicated group of researchers who are concerned about the problems faced by post-stroke individuals.

** This survey can only be taken once by the same IP address.*

Dear Sir/Madam, thank you for taking time out of your busy schedule to follow this study! Your participation will help us understand the health condition of individuals after stroke, so as to carry out targeted improvement studies. Please read the following informed consent carefully. If you agree to participate in our questionnaire, Please check "I agree", then we will ask you some questions.The survey should take about 5 to 10 minutes! Let's get started!

Consent Form

Hello! Thank you very much for taking time to concern this questionnaire. Your information will be important for our study. Before you decide whether to participate in our study, please read the following carefully.

We solemnly promise that this survey study will be anonymous and used only for academic research. If you decide to participate in the study, your personal data during the study will be kept strictly confidential. Any information that can identify you will not be disclosed to others. You can either participate in our study by yourself or help your family members who had a stroke but are unable to complete the online surveys by completing the questionnaire for him/her. Please choose the most appropriate item in each question according to the real situation of you/your family members.

This survey is completely voluntary. If you understand and agree with the above instructions, please check "I agree" in the lower right corner.

National University of Malaysia & Ningxia Medical University

I agree ☐ I disagree ☐

Part A Demographics and clinical characteristics

1. Are you or your family members a target audience (who have experienced the onset of stroke) ?

Myself

My family members（if you choose this item, please note that all the following information are about your family members who have had a stroke）

***2. What is your gender?**

Male

Female

3. What is your age? ☐

4. Which province do you currently live in? ☐

5. Where and with whom do you live?

Home and live with spouse

Home and live with children

Home and live with both spouse and children

Home and live alone

Nursing institution

6. What is your employment status?

Full-time worker (40 or more hours per week)

Part-time worker (less than 40 hours per week)

On leave of absence from work

Not working

Retiree

On disability

7. What is the highest level of education you have completed?

Primary School or below

Junior high school

Senior high school and middle special school

Professional college

University Graduate or above

8. How long have you been diagnosed with stroke?

≤6 months

6 months-1 years

1-2 years

2-5 years

≥5 years

9. What type of stroke have you been diagnosed with?

Brain infarction (atherothrombotic)
Berebral embolism
Cerebral hemorrhage
Subarachnoid hemorrhage
Undetermined

10. What medications did you take long term for recurrent stroke according to doctor's advice?

Antithrombotic: aspirin ☐ clopidogrel ☐ ticagrelor ☐ dipyridamole ☐
aspirin & clopidogrel ☐ aspirin & dipyridamole ☐ other___________
Antilipemic: atorvastatin ☐ rosuvastatin ☐ simvastatin ☐ other____________
Antihypertensive: valsartan ☐ losartan ☐ nifedipine ☐ amlodipine ☐ metoprolol ☐
bisoprolol ☐ hydrochlorothiazide ☐ indapamide ☐ other__________
Antihyperglycemic: metformin ☐ acarbose ☐ voglibose ☐ rosiglitazone ☐ pioglitazone ☐
canagliflozin ☐ glibenclamide ☐ glipizide ☐ gliclazide ☐
gliquidone ☐ glimepiride ☐ other__________
Others:____

11. Did you take antithrombotic medications regularly?

Regularly
Intermittence (discontinued no more than 1 days, for example, miss medications within a day)
Intermittence (discontinued 1-7 days)
Intermittence (discontinued 7-30days)
Intermittence (discontinued 30-60days)
Intermittence (discontinued more than 60 days)
Complete discontinued by yourself
Complete discontinued according to doctor's advice
I have not taken any antithrombotic medications

12. Did you take* antilipemic *medications regularly?

Regularly
Intermittence (discontinued no more than 1 days, for example, miss medications within a day)
Intermittence (discontinued 1-7 days)
Intermittence (discontinued 7-30days)
Intermittence (discontinued 30-60days)
Intermittence (discontinued more than 60 days)
Complete discontinued by yourself
Complete discontinued according to doctor's advice
I have not taken any lipid-lowering medications

13. Did you take antihypertensive medications regularly?

Regularly
Intermittence (discontinued no more than 1 days, for example, miss medications within a day)
Intermittence (discontinued 1-7 days)
Intermittence (discontinued 7-30days)
Intermittence (discontinued 30-60days)
Intermittence (discontinued more than 60 days)
Complete discontinued by yourself
Complete discontinued according to doctor's advice
I have not taken any antihypertensive medications

14. Did you take* antihyperglycemic *medications regularly?

Regularly
Intermittence (discontinued no more than 1 days, for example, miss medications within a day)
Intermittence (discontinued 1-7 days)
Intermittence (discontinued 7-30days)
Intermittence (discontinued 30-60days)
Intermittence (discontinued more than 60 days)
Complete discontinued by yourself
Complete discontinued according to doctor's advice
I have not taken any antihypertensive medications

15. Below is a list of gastrointestinal symptoms. Please select any symptom(s) that you have ever experienced after the onset of stroke, Select all that apply

Difficulty in swallowing (food or liquids sticking in your throat or chest, discomfort with swallowing, or choking sensation when swallowing)
Bowel incontinence (have an accident or soil underclothes)
Heartburn or acid reflux
Bloating or swelling in your belly
Diarrhea (loose, watery, or frequent stools)
Constipation (hard, lumpy, or infrequent stools; straining)
Nausea or vomiting
Gastrointestinal ulcer
Gastrointestinal bleeding
I have not experienced any of these symptoms

16. Below is a list of gastrointestinal symptoms. Please select any symptom(s) that you have ever experienced before the onset of stroke, Select all that apply

Difficulty in swallowing (food or liquids sticking in your throat or chest, discomfort with swallowing, or choking sensation when swallowing)
Bowel incontinence (have an accident or soil underclothes)
Heartburn or acid reflux
Bloating or swelling in your belly
Diarrhea (loose, watery, or frequent stools)
Constipation (hard, lumpy, or infrequent stools; straining)
Nausea or vomiting
Gastrointestinal ulcer
Gastrointestinal bleeding
I have not experienced any of these symptoms since stroke

17. Have you experienced gastrointestinal ulcers or bleeding caused by antithrombotic medications?

Yes, I have experienced before
Yes, I am going through
No, I have never experienced that
I have not taken any antithrombotic drugs

***18. Have you experienced an i*nability to take antithrombotic medications normally due to gastrointestinal ulcers or bleeding?**

Yes, I have experienced before
Yes, I am going through
No, I have never experienced that
I have not taken any antithrombotic drugs

19. You believe that gastrointestinal discomfort caused by a*ntithrombotic *medications (aspirin, clopidogrel, etc.) interferes with compliance with regular secondary prophylaxis.

Strongly disagree
Disagree
Neutral / I don’t know
Agree
Strongly agree

20. Has a healthcare provider ever diagnosed you with any of the following conditions? Select all that apply.

Breast cancer
Colorectal cancer
Esophageal cancer
Liver cancer
Lung cancer
Lymphoma
Pancreatic cancer
Stomach cancer
Throat cancer
Eosinophilic Esophagitis
I have not been diagnosed with any of these conditions

Part B Dysphagia severity measured by NIH PROMIS and its influence on medication intake

If the respondents did not select the dysphagia in question 15 of Part A, the following information is displayed in popup:
Thank you very much for participating in our study. The information you filled in will provide impetus and help us carry out targeted research regarding the health problems of post-stroke patients in the future! Wish you a happy life!
Congratulations, you are qualified for our subsequent survey!

We know from the above survey that you may have dysphagia after stroke. please kindly Go ahead and answer the following survey questions regarding dysphagia, so that we could further understand your feelings and experience about dysphagia. The valuable information you provide will help us to understand the impact of dysphagia on your oral medication, which has guiding significance for us to improve the dosage form of medications for Secondary prophylaxis in the future.

Click the Next button to continue!

1. You mentioned that you had previously experienced trouble in swallowing. Approximately how long ago did you first start having difficulty in swallowing ?

[ ] Year(s) ago [ ] Month(s) ago [ ] day(s) ago

2. In the past 7 days, how often did you experience food getting stuck in your chest when you were eating?

Never
Rarely
Sometimes
Often
Always

3. In the past 7 days, how often did you experience food getting stuck in your throat when you were eating?

Never
Rarely
Sometimes
Often
Always

4. In the past 7 days, how often did you feel pain in your chest when swallowing food?

Never
Rarely
Sometimes
Often
Always

5. In the past 7 days, how often did you have difficulty in swallowing solid foods like meat, chicken or raw vegetables, even after lots of chewing?

Never
Rarely
Sometimes
Often
Always

6. In the past 7 days, how often have you had difficulty in swallowing soft foods like ice cream, apple sauce, or mashed potatoes?

Never
Rarely
Sometimes
Often
Always

7. In the past 7 days, how often have you had difficulty swallowing liquids??

Never
Rarely
Sometimes
Often
Always

8. In the past 7 days, how often have you had difficulty swallowing pills?

Never

Rarely
Sometimes
Often
Always

9. In the past 7 days, how severe was your difficulty swallowing?

Not at all
A little bit
Somewhat
Quite a bit
Very much

10. In the past 7 days, how often did you avoid eating certain foods to prevent trouble in swallowing?

Never
Rarely
Sometimes
Often
Always

11. In the past 7 days, how often did you cut your food into small pieces, or puree or blend your food to avoid trouble in swallowing?

Never
Rarely
Sometimes
Often
Always

12. In the past 7 days, how often did you need a longer time to finish eating your food compared to other people at the table?

Never
Rarely
Sometimes
Often
Always

13. In the past 7 days, how often did you need to drink water or some other liquid to help with swallowing when eating food?

Never
Rarely
Sometimes
Often
Always

14. In the past 7 days, have you take any medication in solid form (e.g. capsules, tablets or pills)?

Yes
No

15. In the past 7 days, how often did you crush or cut your pills, or take liquid forms of medications to avoid trouble in swallowing the pills?

Never
Rarely
Sometimes
Often

Always

16. In the past 7 days, have you had any pain with swallowing?

Yes
No

17. Where 0 is no pain and 10 is the worst pain you could imagine, please rate how severe your pain with swallowing was over the past 7 days?

18. To what extent do you consider difficulty swallowing makes you repulsive to take oral medications?

Not at all
A little bit
Somewhat
Quite a bit
Very much

19. To what extent do you consider difficulty swallowing bring inconvenience in taking oral medications?

Not at all
A little bit
Somewhat
Quite a bit
Very much

20. Have you had any of the following tests to evaluate your difficulty swallowing? Select all that apply.

Upper endoscopy (puts a thin tube with a camera into your mouth and down into your esophagus and stomach)
Barium video swallow (an xray test while you drink a chalky white liquid to examine your throat and upper esophagus)
Barium esophagram (an x ray test while you drink a chalky white liquid to examine your entire esophagus)
Esophageal manometry (a flexible tube is passed through your nose and down into your esophagus, and you take ten sips of water)
I have not had any of these tests
I don't know
Other (please specify)___________

21. Have you confronted a cough when taking oral medications?

Never
Rarely
Sometimes
Often
Always

22. To what extent do you believe your difficulty swallowing was caused by stroke ?

Not at all
A little bit
Somewhat
Quite a bit
Very much

Part C Needs and preference of PSD patients towards preparations of secondary prevention medications

1. How much do you agree that the current secondary prevention medications are easy for you to swallow ??

Strongly disagree
Disagree
Neutral / I don’t know
Agree
Strongly agree

2. How much do you agree that the current secondary prevention medications are suitable for you to take directly (without breaking or crushing tablets)?

Strongly disagree
Disagree
Neutral / I don’t know
Agree
Strongly agree

3. How much do you agree that new oral drug dosage forms easy for swallowing should be developed for secondary prevention medications ?

Strongly disagree
Disagree
Neutral / I don’t know
Agree
Strongly agree

4. Which oral dosage form do you prefer most for secondary prevention medications ?

Oral solid formulation (tablets, capsules,granule formulation,etc.)
Oral liquid formulation (solutions, suspensions,etc.)
Oral semi-solid formulation (oral thick paste, cream, ointment, gels,etc.)

5. How much do you agree that you prefer sustained release preparation to reduce the frequency of secondary prevention medications?

Strongly disagree
Disagree
Neutral / I don’t know
Agree
Strongly agree

6. How much do you agree that you prefer enteric preparation to reduce the risk of gastric adverse reactions (such as ulcer and bleeding)?

Strongly disagree
Disagree
Neutral / I don’t know
Agree

Strongly agree

7. How much do you agree that you prefer combinational preparation of two or more medications to reduce the frequency of secondary prevention medications?

Tablets
Strongly disagree
Disagree
Neutral / I don't know
Agree
Strongly agree

8. To what extent do you believe liquid formulation is more helpful for you to comply with secondary prevention medications as compared to solid ones?

Not at all
A little bit
Somewhat
Quite a bit
Very much

9. To what extent do you believe semi-solid formulation is more helpful for you to comply with secondary prevention medications as compared to solid ones?

Not at all
A little bit
Somewhat
Quite a bit
Very much

10. To what extent do you believe that by taking sustained release preparations will reduce the frequency of administration that is also help you in complying to secondary prevention medications?

Not at all
A little bit
Somewhat
Quite a bit
Very much

11. To what extent do you believe that by taking combinational preparation of two or more drugs will reduce the frequency of administration that is also helpful to comply with secondary prevention medications?

Not at all
A little bit
Somewhat
Quite a bit
Very much

12. Select all factors you consider essential for an appropriate dosage form of secondary prevention medications based on your own experience and preference.

Convenient to carry
Do not need to take with water
The taste is modified with specific flavors(e.g. strawberries, oranges, grapes, etc.)
With smooth texture
Can be bitten or chewed before swallowing(e.g.like jelly)
Easy to swallow

Can be dosed accurately and easily
Do not require reconstituting with water before administration
Can be stored at room temperature
Safe to swallow (avoid aspiration)
Reduced medication frequency
Others (please specify)_____________

***13. Please rank the importance of your selected features according to your own medication experience and preferences.**

	1(not at all)	2	3	4	5(very much)
Convenient to carry	○	○	○	○	○
Do not need to take with water	○	○	○	○	○
The taste is modified with specific flavors	○	○	○	○	○
With smooth texture	○	○	○	○	○
Can be bitten or chewed before swallowing	○	○	○	○	○
Easy to swallow	○	○	○	○	○
Can be dosed accurately and easily	○	○	○	○	○
Do not require reconstituting with water	○	○	○	○	○
Can be stored at room temperature	○	○	○	○	○
Safe to swallow (avoid aspiration)	○	○	○	○	○
Reduced medication frequency	○	○	○	○	○
Others (please specify)_____________	○	○	○	○	○

Part D Perception and acceptability of fluid gels as a appropriate dosage form for PSD patients

To improve compliance to secondary prevention medications among individuals with post-stroke dysphagia (PSD), fluid gels will be further considered to develop into a new dosage form of secondary prevention medications. The next set of questions will be about your perception and acceptability towards fluid gels as a potential disease-specific oral dosage form. Before answering the questions, please read the following statement regarding fluid gels carefully.

Fluid gel *is a soft、jelly-like* ***material*** *that will* ***flow when disturbed*** *(given a spin in the blender or in your mouth)* ***while holding itself together as a solid when at rest.*** *Fluid gel is a new concept posed in recent years and is mainly used in the food industry. The following figures illustrate the rough form of fluid gel and the differences with the conventional liquid forms (solution and suspension).*

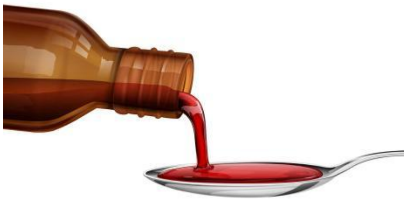

Suspension

https://www.mkmedicine.in/

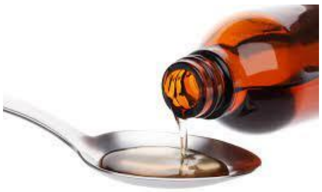

Solution

https://www.mkmedicine.in/

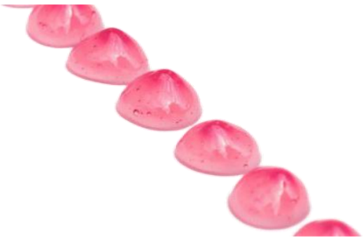

Fluid gel

https://www.chefsteps.com/

1.How much do you agree that you understand what fluid gel is ?

Strongly disagree
Disagree
Neutral / I don't know
Agree
Strongly agree

2. How much do you agree that fluid gel is a promising and appropriate oral drug dosage form for individuals with post-stroke dysphagia?

Strongly disagree
Disagree
Neutral / I don't know
Agree
Strongly agree

3. If fluid gel is developed as usable pharmaceutical formulations, how much do you agree that you prefer fluid gel than the currently available secondary prevention medications ?

Strongly disagree
Disagree
Neutral / I don't know
Agree
Strongly agree

4. How much do you agree that fluid gel is helpful for you to comply with secondary prevention medications?

Strongly disagree

Disagree

Neutral / I don't know

Agree

Strongly agree

5. If the emulsion fluid gel was developed as a new oral drug dosage form for stroke patients with dysphagia What characteristics would you like it to have?

Convenient to carry
Do not need to drink water when and after taking medicines
Can mask drug bitterness
Has smooth texture
Can be bitten or chewed before swallowing
Easy to swallow
Can be dosed accurately and easily

Do not require reconstituting with water before administration
Safe to swallow (avoid aspiration)
Others (please specify)_____________

6. Please rank the importance of your selected features according to your own medication experience and preferences.

	**1(not at all)**	**2**	**3**	**4**	**5(very much)**
Convenient to carry	○	○	○	○	○
Donot need to take with water	○	○	○	○	○
Can mask drug bitterness	○	○	○	○	○
Has smooth texture	○	○	○	○	○
Can be bitten or chewed before swallowing	○	○	○	○	○
Easy to swallow	○	○	○	○	○
Can be dosed accurately and easily	○	○	○	○	○
Do not require reconstituting with water	○	○	○	○	○
Safe to swallow (avoid aspiration)	○	○	○	○	○
Others (please specify)_____________	○	○	○	○	○

7. If the emulsion fluid gel is developed as a new oral medication dosage form for patients with dysphagia after stroke, what is the acceptable single dose volume?

≤5ml
≤10ml
≤15ml
≤20ml
≤30ml
Others_________

8. If the emulsion fluid gel is developed as a new oral medication dosage form for patients with dysphagia after stroke, what would be your acceptable taste?

Milk taste, no bitterness
Fruity, no bitterness
Honey taste, no bitterness
Milk taste, slightly bitter
Fruity, slightly bitter
Honey taste, slightly bitter taste
No bitterness, no special taste
bitter taste

9. If the emulsion fluid gel was developed as a new oral drug dosage form for stroke patients with dysphagia What characteristics would you like it to have?

Convenient to carry
Do not need to drink water when and after taking medicines
Can mask drug bitterness
Has smooth texture
Can be bitten or chewed before swallowing
Easy to swallow
Can be dosed accurately and easily
Do not require reconstituting with water before administration
Safe to swallow (avoid aspiration)
Others (please specify)_____________

10. Please rank the importance of your selected features according to your own medication experience and preferences.

	**1(not at all)**	**2**	**3**	**4**	**5(very much)**

Convenient to carry	○	○	○	○	○
Donot need to take with water	○	○	○	○	○
Can mask drug bitterness	○	○	○	○	○
Has smooth texture	○	○	○	○	○
Can be bitten or chewed before swallowing	○	○	○	○	○
Easy to swallow	○	○	○	○	○
Can be dosed accurately and easily	○	○	○	○	○
Do not require reconstituting with water	○	○	○	○	○
Safe to swallow (avoid aspiration)	○	○	○	○	○
Others (please specify)_____________	○	○	○	○	○

11. Select all possible risks of emulsion fluid gels that you consider as oral drug dosage form for individuals with post-stroke dysphagia?

Inconvenient to carry
Has the risk to cause overdose
More expensive than current solid dosage forms
More expensive than solutions and suspensions
Cannot be stored for a longer time
The volume of dose is excessive
Cannot be dosed accurately and easily
Formulation instability
Cannot be stored at room temperature
Unsafe
Others (please specify)_____________

12. Please rank the severity of your selected features according to your own medication experience and preferences.

	**1(not at all)**	**2**	**3**	**4**	**5(very much)**
Inconvenient to carry	○	○	○	○	○
Has the risk to cause overdose	○	○	○	○	○
More expensive than current solid dosage forms	○	○	○	○	○
More expensive than solutions and suspensions	○	○	○	○	○
Cannot be stored for a longer time	○	○	○	○	○
The volume of dose is excessive	○	○	○	○	○
Cannot be dosed accurately and easily	○	○	○	○	○
Formulation instability	○	○	○	○	○
Can be stored at room temperature	○	○	○	○	○
Unsafe	○	○	○	○	○
Others (please specify)_____________	○	○	○	○	○

Sample size calculation

The sample size of initial target population (individuals who have experienced stroke for not more than 5 years) for this study will be calculated using Raosoft software (http://www.raosoft.com/samplesize.html) by utilizing 5% margin error (E), and 95% of confidence level [Z(c/100)]. The total population size (N) will be set at 20 000 with the assumption that the sample size will not change significantly for populations larger than 20 000[1] . The fraction of

target responses (r) will be set at 50% to obtain the largest sample size possible[2]. The formula for sample size of initial target population (n) is shown below:

$$x=Z(c/100)^2 r(100-r)$$

$$n=N x/((N-1)E^2 + x)$$

According to this formula, the minimum sample size of the initial target population required for this study will be 377. By considering the incidence of PSD is approximately 37-78% and about 50% of them still suffer dysphagia 6 months after the onset of stroke or develop into a persistent dysphagia [3], the calculated minimum sample size for this study will be 967 to 2038.

References

1. Hamburg, M. . (1979). Basic Statistics: A Modern Approach.
2. Gonick, L. , & Smith, W. . (1993). The Cartoon Guide to Statistics [Paperback]. Harper Collins US.
3. Warnecke, T., Labeit, B., Schroeder, J., Reckels, A., Ahring, S., Lapa, S., Claus, I., Muhle, P., Suntrup-Krueger, S. & Dziewas, R. 2021. Neurogenic Dysphagia: Systematic Review and Proposal of a Classification System. *Neurology* 96(6): e876-e889.

Supplementary File 2

Table 1 The compliance of secondary prevention medications among respondents

Medications[a]	All participants (n=3490)	With post-stroke dysphagia Yes (n=1490）	No (n=2000）	P value[b]
**Compliance with Antithrombotic medications (total 3107), n (%)**				
Regularly	2299 (74.0)	1306 (89.6)	993 (60.2)	**<0·0001**
Intermittence (discontinued≤7 days)	706 (22.7)	118 (8.1)	588 (35.7)	
Intermittence (discontinued 8-30days)	72 (2.3)	25 (1.7)	47 (2.8)	
Intermittence (discontinued 31-60days)	31 (1.0)	9 (0.6)	22 (1.3)	
**Compliance with Lipid-Lowering medications(total 1741), n (%)**				
Regularly	1380 (79.3)	872 (80.9)	508 (76.6)	**0.007**
Intermittence (discontinued≤7days)	300 (17.2)	176 (16.3)	124 (18.7)	
intermittence (discontinued 8-30days)	42 (2.4)	25 (2.3)	17 (2.6)	
Intermittence (discontinued 31-60days)	12 (0.7)	5 (0.5)	7 (1.0)	
Intermittence (discontinued＞60days)	6 (0.3)	0 (0.0)	6 (0.9)	
**Compliance with Antihypertensive medications(total 2086), n (%)**				
Regularly	1694 (81.2)	979 (87.6)	715 (73.8)	**<0·0001**
Intermittence (discontinued≤7 days)	341 (16.3)	126 (11.3)	215 (22.2)	
Intermittence (discontinued 8-30days)	38 (1.8)	8 (0.7)	30 (3.1)	
Intermittence (discontinued 31-60days)	12 (0.6)	3 (0.3)	9 (0.9)	
Intermittence (discontinued＞60 days)	1 (0.05)	1 (0.09)	0 (0.0)	
**Compliance with Antidiabetic medications (total 839), n (%)**				
Regularly	640 (76.3)	394 (81.9)	246 (68.7)	**<0·0001**
Intermittence (discontinued≤7 days)	167 (20.0)	77 (16.0)	90 (25.1)	
Intermittence (discontinued 8-30days)	22 (2.6)	6 (1.2)	16 (4.5)	
Intermittence (discontinued 31-60days)	8 (1.0)	3 (0.6)	5 (1.4)	
Intermittence (discontinued＞60 days)	1 (0.1)	1 (0.2)	0 (0)	

[a]Data are presented as n (%); percentages represent the proportion of participants conform to each item among all study sample or the split between groups; statistically significant P values are bolded.

Table 2 Factors associated with compliance of secondary prevention medications

	Adjusted Logistic Regression Models							
	Antithrombotic N=3107		Antilipemic N=1741		Antihypertensive N=2086		Antihyperglycemic N=839	
	OR (95% CI)[c]	P value	OR (95% CI)	P value	OR (95% CI)	P value	OR (95% CI)	P value
**Respondents factors**								
**Gender**								
Male	1 (ref)	..	1 (ref)	..	1 (ref)	..	1 (ref)	..
Female	0.69 (0.59-0.82)	**<0·001**	0.80 (0.62-1.01)	**0.064**	0.91 (0.72-1.14)	0.402	0.62 (0.45-0.87)	**0.005**
**Age, per 10-year increase**	1.34 (1.21-1.49)	**<0·001**	1.07 (0.93-1.24)	0.328	1.11 (0.97-1.28)	0.130	1.12 (0.92-1.36)	0.245

**working status**								
Working (full-time or part time)	1 (ref)	..	1 (ref)	..	1 (ref)	..	1 (ref)	..
On leave of absence from work	1.61 (1.13-2.31)	**0.009**	0.98 (0.56-1.72)	0.937	0.81 (0.50-1.31)	0.399	0.81 (0.38-1.69)	0.569
Not working	1.46 (1.14-1.87)	**0.003**	0.88 (0.58-1.33)	0.537	1.04 (0.73-1.48)	0.823	0.85 (0.50-1.45)	0.554
On disability	5.58 (1.95-16.03)	**0.001**	1.60 (0.53-4.85)	0.407	4.36 (0.57-33.52)	0.157	..	..
**Education level**								
Elementary education or below	1 (ref)	..	1 (ref)	..	1 (ref)	..	1 (ref)	..
Secondary education	1.03 (0.79-1.34)	0.836	0.97 (0.68-1.37)	0.862	0.94 (0.67-1.33)	0.733	1.05 (0.66-1.67)	0.835
Higher education	1.32 (0.97-1.81)	**0.077**	1.26 (0.83-1.93)	0.282	1.00 (0.67-1.51)	0.989	1.67 (0.93-2.98)	0.085
**The course of stroke**								
≤1 years	1 (ref)	..	1 (ref)	..	1 (ref)	..	1 (ref)	..
1-2 years	1.24 (1.02-1.52)	**0.034**	1.09 (0.81-1.47)	0.553	0.81 (0.61-1.09)	0.170	0.79 (0.52-1.21)	0.279
2-5 years	1.57 (1.26-1.95)	**<0·001**	1.24 (0.91-1.71)	0.172	0.79 (0.58-1.07)	0.131	0.95 (0.62-1.45)	0.799
**The subtypes of stroke**								
Ischemic stroke	1 (ref)	..	1 (ref)	..	1 (ref)	..	1 (ref)	..
Hemorrhagic stroke	0.85 (0.69-1.04)	0.115	1.38 (1.06-1.79)	**0.017**	0.96 (0.74-1.23)	0.726	1.11 (0.79-1.59)	0.557
Undetermined	0.43 (0.27-0.70)	**0.001**	1.17 (0.41-3.35)	0.768	0.62 (0.31-1.26)	0.190	0.43 (0.16-1.12)	**0.083**
**with Dysphagia**	5.20 (4.23-6.39)	**<0·001**	1.33 (1.03-1.71)	**0.028**	2.83 (2.21-3.62)	**<0·001**	2.20 (1.56-3.11)	**<0·001**
**Amount of medications taken concurrently**								
≤2 medications	1 (ref)	..	1 (ref)	..	1 (ref)	..	1 (ref)	..
＞2 medications	1.86 (1.57-2.20)	**<0·001**	0.66 (0.49-0.89)	**0.006**	2.09 (1.65-2.66)	**<0·001**	3.14 (1.97-4.99)	**<0·001**

[c]Adjusted multiple logistic regression for the associated of various factors with taking different class of medications regularly were performed. Each factor adjusted for gender, age, working status, education level, course of stroke and subtypes of stroke. Statistically significant P values are bolded.

Table 3 Compensatory maneuvers to cope with dysphagia

Compensatory maneuver performed in the past 7 days	All respondents, n (%) N=1490
**Avoid certain foods to prevent dysphagia**	
Never	64 (4.3)
Rarely	225 (15.1)
Sometimes	632 (42.4)
Often	468 (31.4)
Always	101 (6.8)
**Cut food into small pieces or puree food**	
Never	115 (7.7)
Rarely	293 (19.7)

Sometimes	537 (36.0)
Often	463 (31.1)
Always	82 (5.5)
**Take longer to finish eating food than others**	
Never	0
Rarely	0
Sometimes	220 (14.8)
Often	708 (47.5)
Always	562 (37.7)
**Drink liquid to help with dysphagia**	
Never	39 (2.6)
Rarely	154 (10.3)
Sometimes	362 (24.3)
Often	587 (39.4)
Always	348 (23.4)
**In the past 7 days, have you taken any oral medications?**	
Yes	1478 (99.2)
No	12 (0.8)
**Crush or cut pills or take liquid forms of medicine**	
Never	26 (1.7)
Rarely	139 (9.3)
Sometimes	772 (51.8)
Often	397 (26.6)
Always	156 (10.5)

Table 4 Predictors of Crushing or cutting pills or taking liquid forms of medications

Variable	Crushing or cutting pills or taking liquid forms of medicine, n (%) n=553	OR (95% CI)[c]	P value
**Gender**			
Male	375 (36.8)	1 (ref)	..
Female	178 (37.8)	1.03 (0.78-1.26)	0.827
**Age, per 10-year increase**	61(54,68)	0.96 (0.85-1.10)	0.580
**Employment status**			
Working (full-time or part time)	64 (44.1)	1 (ref)	..
On leave of absence from work	38 (38.4)	0.77 (0.45-1.33)	0.345
Not working	429 (35.6)	0.56 (0.36-0.83)	**0.004**
On disability	22 (55.0)	2.11 (0.98-4.55)	0.056
**Education level**			
Elementary education or below	97 (36.5)	1 (ref)	..
Secondary education	324 (38.9)	1.11 (0.80-1.52)	0.541
Higher education	132 (33.8)	0.84 (0.57-1.24)	0.378
**The course of stroke**			
≤1 years	84 (37.7)	1 (ref)	..
1-2 years	210 (34.7)	0.90 (0.64-1.25)	0.513
2-5 years	259 (39.1)	1.08 (0.78-1.50)	0.641
**The subtypes of stroke**			
Hemorrhagic stroke	175 (38.0)	1 (ref)	..
Ischemic stroke	378 (36.7)	1.03 (0.81-1.30)	0.829
**Amount of medications taken concurrently**			
≤2 medications	101 (28.7)	1 (ref)	..
＞2 medications	452 (43.3)	1.50 (1.14-1.97)	**0.003**
**Dysphagia PROMIS score, Quartile**			
3 (Moderately Symptomatic)	28 (12.8)	1 (ref)	..
4 (Most Symptomatic)	525 (41.7)	4.75 (3.14-7.19)	**<0·0001**

[c]adjusted multiple logistic regression was performed. Each factor adjusted for gender, age, working status, education level, course of stroke, subtypes of stroke, number of medications used and dysphagia PROMIS score in the form of quartile. Statistically significant P values are bolded.

Table 5 Predictors of having a cough when taking oral medications

Variable	having a cough when taking oral medications, n (%) n=351	OR (95% CI)[c]	P value
**Gender**			
Male	375 (36.8)	1 (ref)	..
Female	178 (37.8)	1.23 (0.95-1.59)	0.118
**Age, per 10-year increase**	61(54,68)	0.91 (0.79-1.06)	0.230
**Employment status**			
Working (full-time or part time)	64 (44.1)	1 (ref)	..
On leave of absence from work	38 (38.4)	0.82(0.45-1.48)	0.507
Not working	429 (35.6)	0.85 (0.54-1.33)	0.473
On disability	22 (55.0)	0.98 (0.43-2.23)	0.957
**Education level**			
Elementary education or below	97 (36.5)	1 (ref)	..
Secondary education	324 (38.9)	1.16 (0.80-1.68)	0.449
Higher education	132 (33.8)	1.26 (0.82-1.94)	0.298
**The course of stroke**			
≤1 years	84 (37.7)	1 (ref)	..
1-2 years	210 (34.7)	0.93 (0.64-1.35)	0.712
2-5 years	259 (39.1)	1.16 (0.80-1.66)	0.435
**The subtypes of stroke**			
Hemorrhagic stroke	175 (38.0)	1 (ref)	..
Ischemic stroke	378 (36.7)	0.92 (0.71-1.20)	0.555
**Amount of medications taken concurrently**			
≤2 medications	101 (28.7)	1 (ref)	..
＞2 medications	452 (43.3)	1.12 (0.83-1.51)	0.452
**Dysphagia PROMIS score, Quartile**			
3 (Moderately Symptomatic)	28 (12.8)	1 (ref)	..
4 (Most Symptomatic)	525 (41.7)	3.35 (2.10-5.36)	**<0·0001**

[c]adjusted multiple logistic regression was performed. Each factor adjusted for gender, age, working status, education level, course of stroke, subtypes of stroke, number of medications used and dysphagia PROMIS score in the form of quartile. Statistically significant P values are bolded.

Table 6 Predictors of considering difficulty swallowing makes you repulsive to take oral medications

Variable	consider difficulty swallowing makes you repulsive to take oral medications, n (%) n=1081	OR (95% CI)[c]	P value
**Gender**			
Male	375 (36.8)	1 (ref)	..
Female	178 (37.8)	1.12 (0.87-1.45)	0.372
**Age, per 10-year increase**	61(54,68)	0.84 (0.73-0.97)	**0.019**
**Employment status**			
Working (full-time or part time)	64 (44.1)	1 (ref)	..

On leave of absence from work	38 (38.4)	1.36 (0.76-2.43)	0.296
Not working	429 (35.6)	1.59 (1.02-2.48)	**0.039**
On disability	22 (55.0)	2.60 (1.04-6.47)	**0.040**
**Education level**			
Elementary education or below	97 (36.5)	1 (ref)	..
Secondary education	324 (38.9)	0.93 (0.66-1.30)	0.664
Higher education	132 (33.8)	1.05 (0.70-1.60)	0.808
**The course of stroke**			
≤1 years	84 (37.7)	1 (ref)	..
1-2 years	210 (34.7)	0.97 (0.68-1.38)	0.846
2-5 years	259 (39.1)	1.11 (0.78-1.58)	0.560
**The subtypes of stroke**			
Hemorrhagic stroke	175 (38.0)	1 (ref)	..
Ischemic stroke	378 (36.7)	1.29 (1.00-1.66)	**0.046**
**Amount of medications taken concurrently**			
≤2 medications	101 (28.7)	1 (ref)	..
＞2 medications	452 (43.3)	1.15 (0.87-1.51)	0.332
**Dysphagia PROMIS score, Quartile**			
3 (Moderately Symptomatic)	28 (12.8)	1 (ref)	..
4 (Most Symptomatic)	525 (41.7)	3.82 (2.83-5.16)	**<0·0001**

[c]adjusted multiple logistic regression was performed. Each factor adjusted for gender, age, working status, education level, course of stroke, subtypes of stroke, number of medications used and dysphagia PROMIS score in the form of quartile. Statistically significant P values are bolded.

Table 7 Predictors of consider difficulty swallowing bring inconvenience in taking oral medications

Variable	consider difficulty swallowing bring inconvenience in taking oral medications, n (%) n=825	OR (95% CI)[c]	P value
**Gender**			
Male	375 (36.8)	1 (ref)	..
Female	178 (37.8)	1.06 (0.85-1.32)	0.614
**Age, per 10-year increase**	61(54,68)	1.07 (0.94-1.21)	0.299
**Employment status**			
Working (full-time or part time)	64 (44.1)	1 (ref)	..
On leave of absence from work	38 (38.4)	0.77 (0.46-1.29)	0.320
Not working	429 (35.6)	0.77 (0.51-1.14)	0.193
On disability	22 (55.0)	0.81 (0.40-1.66)	0.569
**Education level**			
Elementary education or below	97 (36.5)	1 (ref)	..
Secondary education	324 (38.9)	1.04 (0.77-1.41)	0.790
Higher education	132 (33.8)	1.29 (0.90-1.86)	0.167
**The course of stroke**			
≤1 years	84 (37.7)	1 (ref)	..
1-2 years	210 (34.7)	0.97 (0.71-1.33)	0.846
2-5 years	259 (39.1)	1.24 (0.91-1.69)	0.176
**The subtypes of stroke**			
Hemorrhagic stroke	175 (38.0)	1 (ref)	..
Ischemic stroke	378 (36.7)	1.25 (1.00-1.56)	0.051
**Amount of medications taken concurrently**			
≤2 medications	101 (28.7)	1 (ref)	..
＞2 medications	452 (43.3)	0.88 (0.69-1.13)	0.321

**Dysphagia PROMIS score, Quartile**			
3 (Moderately Symptomatic)	28 (12.8)	1 (ref)	..
4 (Most Symptomatic)	525 (41.7)	1.85 (1.38-2.49)	**<0·0001**

[c]adjusted multiple logistic regression was performed. Each factor adjusted for gender, age, working status, education level, course of stroke, subtypes of stroke, number of medications used and dysphagia PROMIS score in the form of quartile. Statistically significant P values are bolded.
